# Mitral valve posteromedial papillary muscle head rupture presenting after liposomal doxorubicin chemotherapy for metastatic lung liposarcoma: a case report

**DOI:** 10.1093/ehjcr/ytag566

**Published:** 2026-07-30

**Authors:** Shivam U Desai, Narayana R Gandham

**Affiliations:** Department of Internal Medicine, Corewell Health South, 1234 Napier Avenue, St. Joseph, MI 49085, USA; Department of Internal Medicine, Corewell Health South, 1234 Napier Avenue, St. Joseph, MI 49085, USA

**Keywords:** Anthracycline cardiotoxicity, Papillary muscle rupture, Acute severe mitral regurgitation, Cardio-oncology, Case report

## Abstract

**Background:**

Doxorubicin-induced cardiotoxicity classically manifests as dose-dependent left ventricular systolic dysfunction. Herein, we describe an unusual case of acute mitral papillary muscle rupture following cumulative anthracycline therapy, presenting despite a preserved ejection fraction.

**Case summary:**

A 78-year-old woman with metastatic liposarcoma and multiple cardiovascular risk factors developed acute dyspnoea 1 week after completing liposomal doxorubicin (cumulative absolute dose 534 mg). She previously received 5-fraction stereotactic body radiation therapy to the lower lung lobes in September 2024 (9 months prior), targeting fields in close geographic proximity to the diaphragmatic and inferior heart walls. Surveillance echocardiograms from 2022 to 2025 showed a preserved left ventricular ejection fraction (65%–75%). At presentation, cardiac biomarkers showed a normal brain natriuretic peptide and flat, minimally elevated troponins. Transoesophageal echocardiography confirmed an acute rupture of the posteromedial papillary muscle head and associated chordae causing severe (4+) mitral regurgitation. Open surgical intervention was deferred due to advanced malignancy and goals of care. Her acute heart failure was optimized medically with intravenous loop diuretics, low-dose lisinopril for afterload reduction, and metoprolol succinate, while home amlodipine and hydrochlorothiazide were discontinued. She stabilized and is undergoing evaluation for transcatheter edge-to-edge repair.

**Discussion:**

While a direct causal link is unproven, this case highlights a rare structural complication. We present this ‘two-hit’ model as a hypothesis-generating observation, suggesting that chemotherapy-induced structural changes might compound underlying ischaemic or radiation-induced vulnerabilities. As such, advanced imaging modalities may offer value in future efforts to implement risk-adapted surveillance.

Learning points

- Anthracycline toxicity can rarely manifest as acute, subvalvular mechanical failure, such as papillary muscle rupture, despite a preserved left ventricular ejection fraction.
- Clinicians should be aware that thoracic radiation may interact with anthracyclines, compounding cardiotoxicity and accelerating localized tissue vulnerability.

## Introduction

Doxorubicin is an anthracycline commonly used to treat sarcomas, breast cancer, and haematological malignancies.^1^ Its antineoplastic effects result from DNA intercalation, topoisomerase II inhibition, and reactive oxygen species generation.^2^

Anthracycline-induced cardiotoxicity is well established and commonly manifests as dose-dependent cardiomyopathy and congestive heart failure.^2,3^ Proposed mechanisms include oxidative stress, mitochondrial dysfunction, and cardiomyocyte apoptosis, leading to diffuse myocardial injury and adverse remodelling.^3^

Although systolic dysfunction is the hallmark of anthracycline cardiotoxicity, acute structural subvalvular complications such as papillary muscle rupture have not been described in published literature. We report a case of severe mitral regurgitation secondary to an acute rupture of the posteromedial papillary muscle head with associated chordal disruption and a flail posterior mitral leaflet, presenting shortly after completion of liposomal doxorubicin therapy. While traditional ischaemic aetiologies remain the primary driver of such mechanical failures, this case highlights a striking temporal association with recent anthracycline exposure, underscoring the need for heightened vigilance regarding atypical structural complications.

## Summary figure

**Figure:**
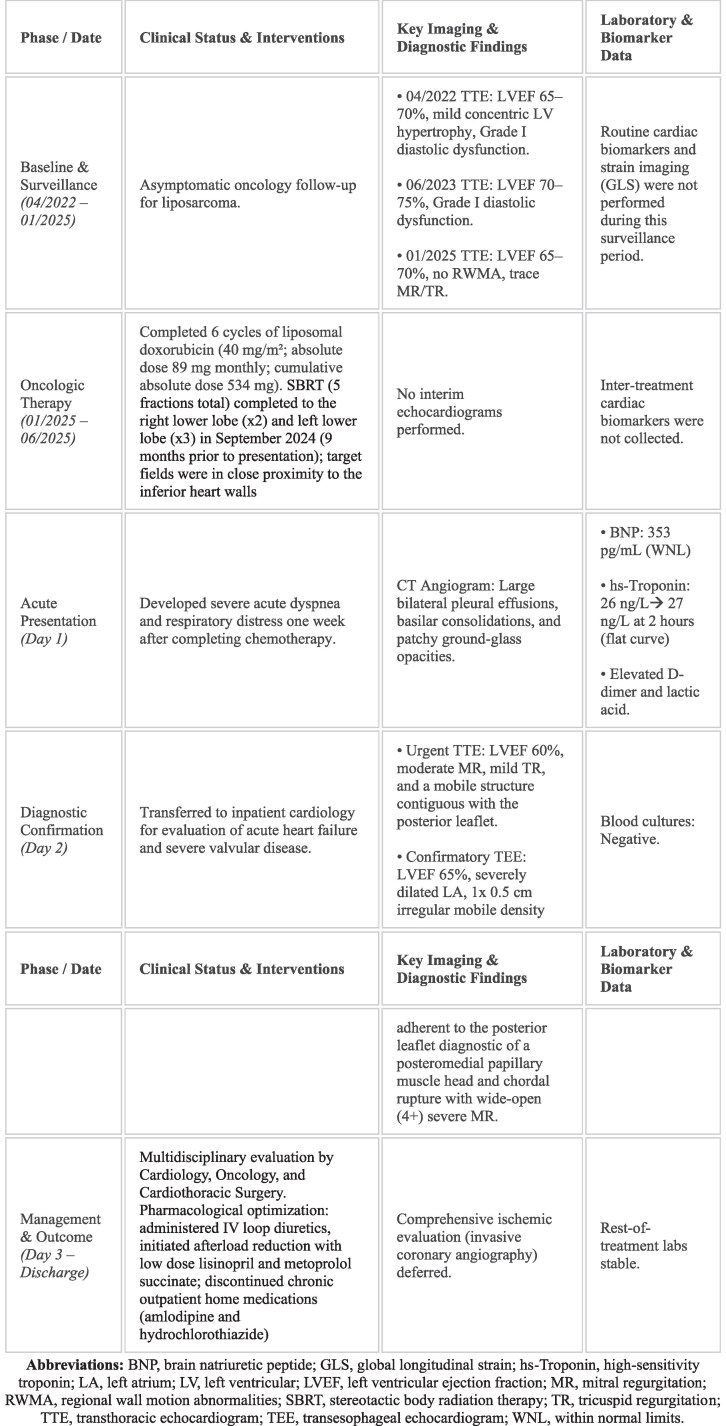


## Case presentation

**Figure 1:**
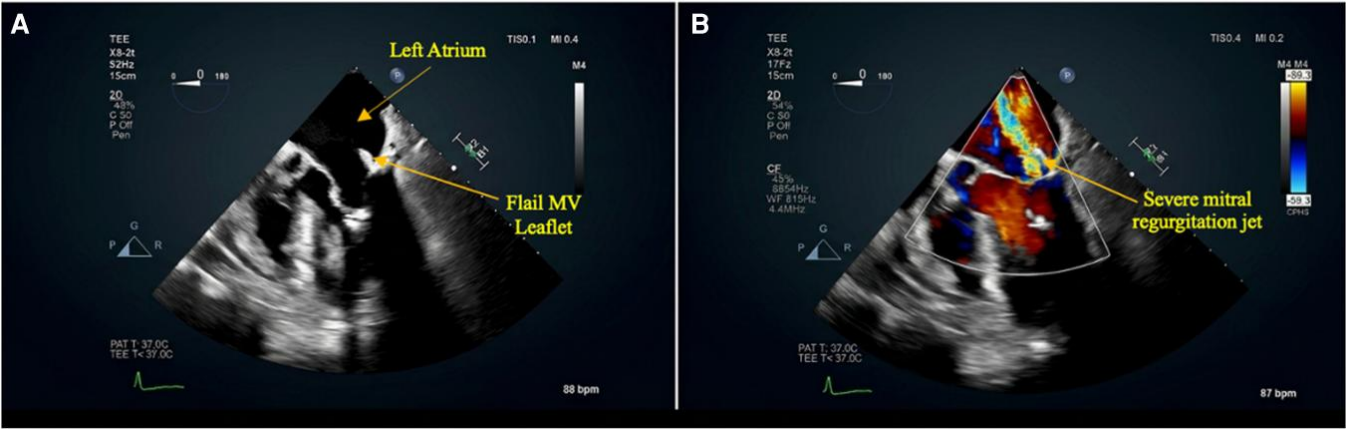
Transoesophageal echocardiographic assessment of acute mechanical valvular failure. (*A*) 3D transoesophageal echocardiogram (mid-oesophageal view) demonstrating acute structural collapse of the subvalvular apparatus. The arrowhead highlights a distinct flail posterior mitral valve leaflet prolapsing retrogradely into a dilated left atrium, secondary to an acute rupture of the posteromedial papillary muscle head. (*B*) Transoesophageal echocardiogram with colour Doppler evaluation over the identical mid-oesophageal plane. The colour flow mapping reveals a massive, highly eccentric, high-velocity jet filling much of the left atrium, confirming severe (4+) acute mitral regurgitation resulting from the subvalvular mechanical failure.

A 78-year-old woman with a history of obesity, type 2 diabetes, hyperlipidaemia, hypertension, and metastatic dedifferentiated lung liposarcoma presented with progressive dyspnoea and orthopnoea for 3 days. Following resection of a primary spindle cell carcinoma, surveillance computed tomography imaging 18 months later confirmed metastatic dedifferentiated liposarcoma via nodule biopsy. She received five-fraction stereotactic body radiation therapy (SBRT) to the right lower lobe (two sessions) and left lower lobe (three sessions) completed in September 2024 (9 months prior to presentation). Given the lower lobe targets, these radiation fields were in close geographic proximity to the diaphragmatic and inferior surfaces of the heart. Following SBRT, she received six cycles of single-agent liposomal doxorubicin (40 mg/m^2^ monthly based on a 2.23 m^2^ body surface area), yielding an absolute monthly dose of 89 mg and a cumulative dose of 534 mg over 6 months. She had no prior exposure to free doxorubicin or other chemotherapeutic agents. One week after her final cycle, she presented with acute dyspnoea (*Figure 1*).

Upon arrival, she was tachypnoeic to 93% on room air with physical examination notable for rales and bilateral pedal oedema. Laboratory evaluation revealed elevated D-dimer and lactic acid levels. Cardiac biomarkers demonstrated a normal brain natriuretic peptide of 353 pg/mL (normal reference range <100 pg/mL) and flat, minimally elevated high-sensitivity troponin levels of 26 ng/L, trending to 27 ng/L (normal reference range <14 ng/L).

During her 5-day admission, her acute heart failure was managed with a carefully tailored medical regimen. She was optimized with intravenous loop diuretics (furosemide) for decongestion and initiated on low-dose lisinopril to provide targeted afterload reduction, essential in mitigating the haemodynamic stress of severe mitral regurgitation—alongside metoprolol succinate. To prevent worsening hypotension or competing therapeutic effects, her home antihypertensive agents, amlodipine, and hydrochlorothiazide, were discontinued. This approach resulted in rapid resolution of her oxygen requirement and volume stabilization.

Given her advanced metastatic malignancy and an oncologic life expectancy estimated at less than 5 years, a multidisciplinary heart team determined that she was not a candidate for traditional open-heart surgical valve repair or replacement. Concurrently, given her flat troponin curve and the choice of palliative medical management, invasive coronary angiography was deferred. This approach aligned with the patient’s explicit goals of care, which prioritized quality of life and end-of-life preparation over high-risk surgery. She was transitioned to oral medications and discharged. To optimize her severe valvular disease within her goals of care, she is currently undergoing evaluation at a tertiary centre for a less invasive transcatheter edge-to-edge repair using a MitraClip. At her subsequent follow-up, she remains alive and clinically stable on medical therapy without any recurrent cardiac rehospitalizations.

## Discussion

The patient’s presentation was consistent with acute mechanical mitral regurgitation. The onset of symptoms 1-week post-chemotherapy highlights a striking temporal proximity to expected anthracycline-associated myocardial remodelling. Although anthracycline toxicity most commonly manifests as progressive systolic dysfunction, serial transthoracic echocardiograms showed a gradual decline in left ventricular ejection fraction (LVEF) from 70% to 75% to only 60% over 3 years. Transoesophageal echocardiography subsequently demonstrated severe mitral regurgitation due to a rupture of the posteromedial papillary muscle head with associated chordal disruption and a flail posterior mitral leaflet despite preserved systolic function, highlighting its potential to precipitate rare mechanical complications.

Papillary muscle rupture typically necessitates rapid diagnosis and urgent surgical correction. However, in this instance, surgical mitral valve replacement was not pursued due to the patient’s limited life expectancy and overall poor prognosis. The risks of operative intervention were deemed to outweigh the potential therapeutic benefit, and a medically focused approach was adopted.

The omission of invasive coronary angiography or cardiac magnetic resonance imaging (MRI) prevents the absolute exclusion of an underlying ischaemic aetiology. Given her multiple cardiovascular risk factors, including advanced age, type 2 diabetes, hypertension, and hyperlipidaemia, localized papillary muscle ischaemia or a microvascular infarction remains a highly plausible driver of mechanical subvalvular failure. Clinically, the absence of acute ST-segment changes on electrocardiogram (ECG), the lack of regional wall motion abnormalities, and flat, low-level troponins collectively argued against a large epicardial acute myocardial infarction as the instigating event. However, these findings alone cannot rule out subclinical coronary artery disease or localized microvascular ischaemic necrosis. Ultimately, the decision to forgo invasive anatomical mapping was driven by the patient’s critical valvular status, advanced metastatic malignancy, and explicit goals of care. Concurrently, the five-fraction scattered radiation from her right and left lower-lobe SBRT, completed 9 months prior in September 2024, could have contributed to microvascular changes or localized tissue vulnerability due to its close geographic proximity to the inferior cardiac walls.

Other retrospective limitations include the lack of intermediate surveillance cardiac biomarkers and baseline speckle-tracking strain metrics, which limits our ability to evaluate whether subclinical microstructural or subvalvular strain anomalies preceded the final mechanical disruption.

However, the striking temporal proximity to her completion of cumulative anthracycline therapy raises the compelling possibility of a ‘two-hit’ mechanism, wherein subclinical chemotherapy-induced structural remodelling may have compounded underlying ischaemic vulnerability to precipitate this subvalvular failure. Importantly, we present this ‘two-hit’ model strictly as a hypothesis-generating observation rather than an established, definitive pathophysiological pathway. Given the lack of existing literature on isolated papillary muscle rupture directly resulting from anthracycline use, this framework serves to stimulate further translational exploration into how chemotherapeutic tissue remodelling might interact with traditional cardiovascular risk factors to provoke unexpected subvalvular structural collapse.

This case underscores an important clinical awareness gap in the cardiovascular care of patients receiving anthracycline therapy. While current surveillance strategies focused on detecting left ventricular systolic dysfunction are well established and generally appropriate, rare structural or mechanical complications may remain under-recognized due to their infrequency. Recognition of such potential presentations broadens the spectrum of suspected anthracycline-associated cardiac injury and highlights an area that warrants heightened clinical vigilance rather than intensified routine monitoring. Standard monitoring relying solely on periodic LVEF assessments can fail to detect subtle structural abnormalities preceding overt mechanical failure.^4^ A comprehensive surveillance strategy, such as those proposed by the European Society of Cardiology, involves regular 12-lead ECG, transthoracic echocardiography, and cardiac biomarkers based on baseline risk stratification to aid in early detection.^5^ This case highlights the clinical value of reporting such atypical mechanical complications. Furthermore, future research should investigate whether advanced imaging modalities, such as strain echocardiography and cardiac MRI, can be effectively utilized as hypothesis-testing tools to identify patients at heightened risk for structural failure before catastrophic events occur.^6,7^

## Data Availability

The data underlying this article are available in the article and can be shared on reasonable request to the corresponding author.

## Lead author biography

Shivam Desai, MD, MS, is a first-year Internal Medicine resident at Corewell Health South in St. Joseph, Michigan. Originally from Connecticut, he completed his undergraduate degree at the University of Connecticut and earned his medical degree from Central Michigan University College of Medicine. His cardiology interests include electrophysiology, heart failure, sports cardiology, and cardiovascular prevention. He is passionate about advancing innovation through research and plans to pursue a cardiovascular fellowship with a focus on academic medicine and patient-centred care.

## Notes

**Consent:** The authors confirm that written, informed consent was obtained from the patient for the publication of this case report, including all diagnostic transoesophageal echocardiography images, timeline records, and associated clinical data, in accordance with the Committee on Publication Ethics (COPE) guidelines. Institutional review board approval was waived in accordance with institutional policies governing anonymous case reports.

## References

[1] Carvalho C, Santos RX, Cardoso S, Correia S, Oliveira PJ, Santos MS, et al Doxorubicin: The good, the bad and the ugly effect. Curr Med Chem 2009;16:3267–3285. 19548866 10.2174/092986709788803312

[2] Kong CY, Guo Z, Song P, Zhang X, Yuan YP, Teng T, et al Underlying the mechanisms of doxorubicin-induced acute cardiotoxicity: oxidative stress and cell death. Int J Biol Sci 2022;18:760–770. 35002523 10.7150/ijbs.65258 PMC8741835

[3] Linders AN, Dias IB, López Fernández T, Tocchetti CG, Bomer N, Van der Meer P. A review of the pathophysiological mechanisms of doxorubicin-induced cardiotoxicity and aging. npj Aging 2024;10:9. 38263284 10.1038/s41514-024-00135-7 PMC10806194

[4] Chaulin AM . The essential strategies to mitigate cardiotoxicity caused by doxorubicin. Life (Basel) 2023;13:2148. 38004288 10.3390/life13112148 PMC10672543

[5] Lyon AR, López-Fernández T, Couch LS, Asteggiano R, Aznar MC, Bergler-Klein J, et al 2022 ESC Guidelines on cardio-oncology developed in collaboration with the European Hematology Association (EHA), the European Society for Therapeutic Radiology and Oncology (ESTRO) and the International Cardio-Oncology Society (IC-OS). Eur Heart J 2022;43:4229–4361. 36017568 10.1093/eurheartj/ehac244

[6] Edvardsen T, Asch FM, Davidson B, Delgado V, DeMaria A, Dilsizian V, et al Non-invasive imaging in coronary syndromes: recommendations of The European Association of Cardiovascular Imaging and the American Society of Echocardiography, in Collaboration with The American Society of Nuclear Cardiology, Society of Cardiovascular Computed Tomography, and Society for Cardiovascular Magnetic Resonance. J Am Soc Echocardiogr 2022;35:329–354. 35379446 10.1016/j.echo.2021.12.012

[7] De Lazzari M, Cipriani A, Cecere A, De Gaspari M, Graziano F, Nazzi M, et al Cardiac rupture in acute myocardial infarction: a cardiac magnetic resonance study. Eur Heart J Cardiovasc Imaging 2023;24:1491–1500. 37200615 10.1093/ehjci/jead088 PMC10610764

